# Poster Session I - A56 DOUBLE-BALLOON-ERCP IN POST-LIVER TRANSPLANT PATIENTS WITH ROUX EN Y HEPATICOJEJUNOSTOMY: ANALYSIS OF OUTCOMES IN A SINGLE CANADIAN CENTRE

**DOI:** 10.1093/jcag/gwaf042.056

**Published:** 2026-02-13

**Authors:** J Soleas, R Mackay, S Wasilenko, B Halloran, S Zepeda Gomez

**Affiliations:** Gastroenterology, University of Alberta, Edmonton, AB, Canada; Gastroenterology, University of Alberta, Edmonton, AB, Canada; Gastroenterology, University of Alberta, Edmonton, AB, Canada; Gastroenterology, University of Alberta, Edmonton, AB, Canada; Gastroenterology, University of Alberta, Edmonton, AB, Canada

## Abstract

**Background:**

Double-balloon enteroscopy-assisted endoscopic retrograde cholangiopancreatography (DBE-ERCP) is a complex procedure that allows for biliary interventions in patients with surgically altered anatomy. Around 10% of patients with liver transplant will undergo a Roux-en-Y hepaticojejunostomy. The University of Alberta Hospital is a reference centre for liver transplant and advanced therapeutic endoscopy.

**Aims:**

The aim of this study is to review the experience and analyze the outcomes of post-liver transplant patients with Roux-en-Y hepaticojejunostomy anatomy who have undergone DBE-ERCP.

**Methods:**

Retrospective analysis with data collected from all DBE-ERCP procedures performed from July 2011- July 2025 at the University of Alberta Hospital.

**Results:**

One hundred and fifteen patients underwent DBE-ERCP, of those, sixty-seven were performed on patients following orthotopic liver transplantation with Roux-en-Y hepaticojejunostomy. The indications for DBE-ERCP were as follows: 51 (78%) patients presented with abnormal liver enzymes; 48 (73%) patients had associated abnormal imaging findings; 28 (42%) presented with cholangitis; and 3 (4%) were suspected of having choledocholithiasis. The overall technical success was 84% (56/67) as defined by reaching the hepaticojejunostomy with therapeutic interventions; of these 56 patients, 41 (73%) were found to have a stricture at the anastomosis and 37 (90%) underwent balloon dilation. Eighteen patients (43%) had stricture recurrence and required repeat DBE-ERCP. Median time to repeat DBE-ERCP was 5 months. From these patients, eleven (61%) underwent repeat dilation, 6 (33%) underwent both dilation and stent placement, and five (28%) required PTC placement. Three patients required re-transplantation as a result of recurrent cholangitis. Nine (50%) patients who experienced re-stenosis of the hepaticojejunostomy required a third DBE-ERCP. The average time to the third DBE-ERCP was 6.4 months. One patient procedure was complicated by mild bleeding requiring stent placement, and one patient had a micro-perforation after balloon dilation, which was managed with PTC placement. Within the group who had stricture recurrence, 5 patients (28%) had documented hepatic artery stenosis.

**Conclusions:**

DBE-ERCP is a safe and effective procedure for the management of biliary obstruction in patients with Roux-en-Y hepatico-jejunostomy anatomy after orthotopic liver transplant. Stricture formation is common and recurrence happens in up to 40% of cases, particularly in patients with associated hepatic artery stenosis.

**Funding Agencies:**

None

